# FORTIFYING HOPE: OLDER LATINA IMMIGRANTS WITH CANCER

**DOI:** 10.1093/geroni/igae098.0993

**Published:** 2024-12-31

**Authors:** Iraida Carrion, Malinee Neelamegam, Tania Estapé, Jorge Estapé

**Affiliations:** University of South Florida, Tampa, Florida, United States; University of North Texas Health Science Center, Fort Worth, Texas, United States; Fundación Contra El Cáncer, Barcelona, Catalonia, Spain; Fundación Contra El Cáncer, Barcelona, Catalonia, Spain

## Abstract

Given the growing population of Latina immigrants aged 60 years and older in Central Florida, United States, and the current lack of relevant data, understanding this population’s cancer experiences is crucial to ensure effective interventions, psychosocial care, and policies. This study assessed 200 responses to a survey developed and administered in Spanish, with questions focused on cancer knowledge, attitudes, prevention, early diagnosis, and treatment. The survey included a qualitative component of open-ended questions regarding the perspectives and experiences of 23 older immigrant Latina women with cancer. Recruitment occurred in community-based settings and the interviews were transcribed in Spanish and English. Utilizing a grounded theory approach and thematic analysis, codes were developed. The survey data highlight the lack of initial information regarding available cancer treatments, a minimal understanding of the causes of cancer, and insufficient knowledge regarding cancer diagnoses that delayed early treatment. Within this sample, 52% had breast cancer, 22% had skin cancer, 13% had uterine cancer, 4% had colon cancer, and 4% had uterine and ovarian cancer. Latina cancer rates are expected to rise in the next 20 years. Promoting cancer screenings and modifying health-related behaviors among diverse older immigrant Latinas is imperative and requires the development of culturally appropriate interventions to overcome health disparities and treatment barriers.

